# Insights into transportation CO_2_ emissions with big data and artificial intelligence

**DOI:** 10.1016/j.patter.2025.101186

**Published:** 2025-03-03

**Authors:** Zhenyu Luo, Tingkun He, Zhaofeng Lv, Junchao Zhao, Zhining Zhang, Yongyue Wang, Wen Yi, Shangshang Lu, Kebin He, Huan Liu

**Affiliations:** 1 State Key Joint Laboratory of ESPC, School of Environment, Tsinghua University, Beijing 100084, China; 2 International Joint Laboratory on Low Carbon Clean Energy Innovation, Ministry of Education, Beijing, China

**Keywords:** carbon neutrality, transportation emissions, intelligent transportation system, big data, artificial intelligence

## Abstract

The ever-increasing stream of big data offers potential for deep decarbonization in the transportation sector but also presents challenges in extracting interpretable insights due to its complexity and volume. This overview discusses the application of transportation big data to help understand carbon dioxide emissions and introduces how artificial intelligence models, including machine learning (ML) and deep learning (DL), are used to assimilate and understand these data. We suggest using ML to interpret low-dimensional data and DL to enhance the predictability of data with spatial connections across multiple timescales. Overcoming challenges related to algorithms, data, and computation requires interdisciplinary collaboration on both technology and data.

## The bigger picture

The transportation sector accounts for one-fifth of global CO_2_ emissions and is one of the most challenging sectors to decarbonize due to its heavy reliance on fossil fuels. As urbanization and economic development accelerate, a critical question arises: how can we effectively decarbonize transportation while sustaining economic growth? A significant barrier is the limited understanding of real-world transportation CO_2_ emissions, especially their dynamic and localized characteristics, which impedes the development of targeted and effective mitigation strategies. In this era of information explosion, big data and artificial intelligence (AI) hold significant potential to enhance our understanding of transportation emissions. As the global community works to meet the ambitious goals of the Paris Agreement and achieve carbon neutrality, integrating data-driven insights into policy and decision-making becomes increasingly crucial.

This perspective highlights a novel toolkit to comprehend transportation emissions through AI-powered analysis of big data, demonstrating how advanced technologies bridge the gap between scientific innovation and practical solutions. The ability to extract and apply meaningful knowledge from complex data will be indispensable for governments and industries alike, offering a valuable contribution to the global efforts toward deep transportation decarbonization. Meanwhile, such applications present significant challenges and opportunities for data and AI technologies, emphasizing the importance of cross-disciplinary collaboration to foster technological advancements and data sharing, while paving the way for smarter, more efficient pathways toward carbon neutrality.

## Graphical abstract

**Figure:**
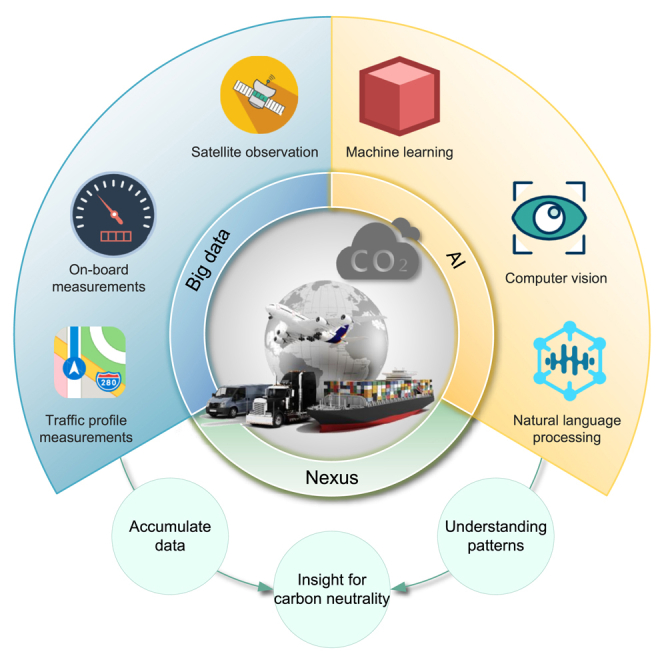

## Teaser

Big data show significant potential for deep decarbonization in the transportation sector, while advances in artificial intelligence (AI) technologies help extract valuable knowledge from such data. This perspective reviews the applications of big data and AI technologies in transportation, encompassing data accumulation, pattern understanding, and the intricate nexus between transportation activities and carbon emissions, ultimately providing insights for achieving carbon neutrality.

## Introduction

The Intergovernmental Panel on Climate Change reported that global greenhouse gas (GHG) emissions reached unprecedented levels from 2010 to 2019, underscoring the urgent need for reductions across all sectors.^1^ The transportation sector, including aviation, shipping, and on-road vehicles, significantly contributes to economic growth but accounts for 20% of global carbon dioxide (CO_2_) emissions.^2^ With the expansion of emerging economies, transportation emissions are outpacing most other industries and are projected to double by 2050.^3^ However, decarbonizing the transportation sector remains particularly challenging due to its dependence on fossil fuels, raising a critical question: does transportation hinder future CO_2_ emission reduction efforts?

Achieving carbon-neutral transportation necessitates a comprehensive understanding of CO_2_ emissions. However, the sector’s dynamic nature introduces significant spatiotemporal variability, complicating emission accounting.^4^ While macro-level data, such as statistical yearbooks, are commonly used for estimating transportation emissions,^5^^,^^6^ this method often misrepresents spatiotemporal distributions, particularly in developing nations where data may be incomplete or unreliable. Over the past decade, advances in environmental informatics have greatly improved emission analysis and applications.^7^ Data-driven policies play a critical role in enabling countries to meet their Paris Agreement commitments and reach peak carbon emissions. Big data refer to vast, complex, and continuously expanding datasets from diverse sources.^8^ In transportation, such data span various scales, including roadside sampling (on-road remote sensing), environmental monitoring at tens of meters, and satellite observations covering hundreds of kilometers. The Internet of Things (IoT) advances urban intelligent transportation systems (ITSs),^9^ enabling the daily transmission of vast transportation data.^10^ This innovation offers a significant opportunity to address policy assessment challenges and drive deep decarbonization.

**Figure 1:**
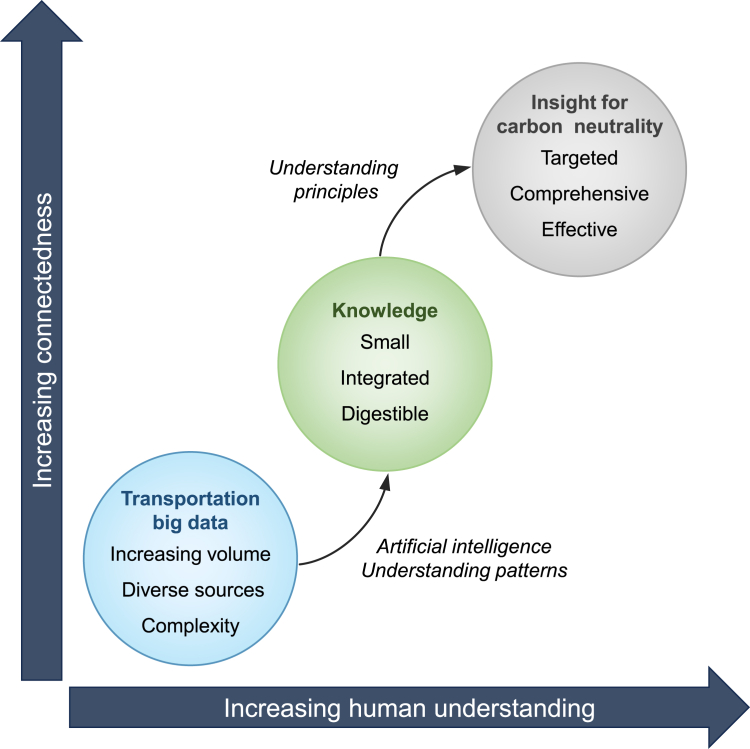
The cascade of transportation big data, knowledge, and insight for carbon-neutral transportation

The era of big data generates massive volumes of transportation data daily, with storage exceeding petabytes and transmission rates reaching hundreds of terabytes per day.^11^ However, data collection outpaces our ability to process and analyze it effectively. The key challenge lies in extracting valuable insights from these datasets and integrating them across disciplines (Figure 1). The emergence of novel data sources has driven advances in computing, while recent progress in artificial intelligence (AI) offers new frameworks for analyzing transportation big data.^12^ Machine learning (ML) efficiently processes large, high-dimensional datasets with relatively simple structures.^13^ By contrast, deep learning (DL)^14^ excels in capturing complex spatial and temporal patterns that influence system behavior.^15^

The subsequent sections examine transportation big data and their role in analyzing CO_2_ emissions. We explore the application of ML in processing such data and emphasize DL’s potential to address their increasing complexity. Finally, we discuss challenges and emerging opportunities at the intersection of AI and transportation research.

## Transportation big data

### On-board measurements

**Figure 2:**
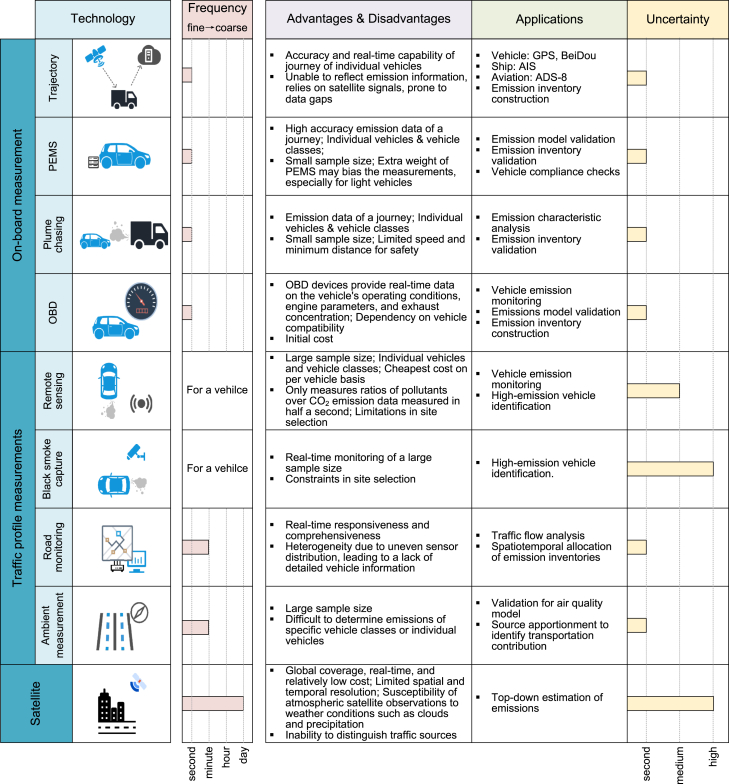
Major existing transportation big data

Real-time operational data from vehicles, ships, and aircraft are recorded through sensors and modules, ensuring safety, efficiency, and accuracy (Figure 2). These measurements are crucial for analyzing emission patterns, such as their spatiotemporal distribution and characteristics.

**Figure 3:**
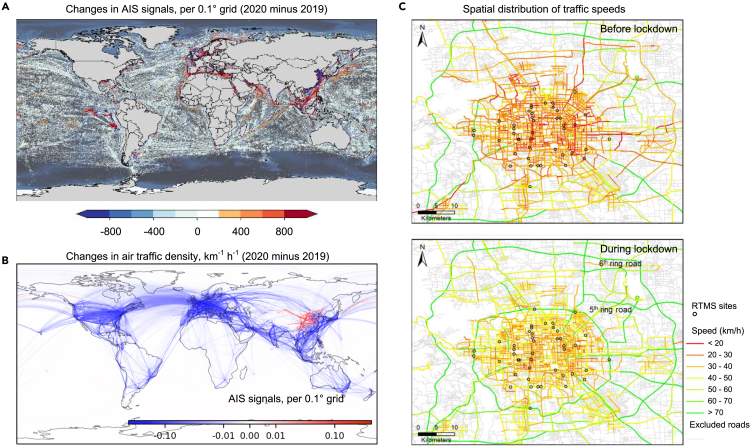
Applications of big data for characterizing the transportation activity changes Examples of (A) ships,^27^ (B) aircraft,^26^ and (C) vehicles under the impact of COVID-19. (C) reprinted with permission from Lv et al.^28^ Copyright 2025 American Chemical Society.

Trajectory data, commonly gathered using the global positioning system (GPS) or similar location sensors, are widely used to track travel paths as well as operational factors, including speed and direction. GPS is frequently deployed in on-road transportation, particularly in taxis and buses, due to its low cost.^16^ Due to limited vehicle deployment and activity range, trajectory data are often used to allocate aggregate emissions across road networks.^17^^,^^18^^,^^19^ Recently, GPS data have helped assess the carbon emission benefits of electrification or fuel transition for passenger vehicles.^20^ The BeiDou navigation satellite system, which tracks the speed and location of heavy-duty trucks (HDTs),^9^ offers near-real-time, high-coverage data for billions of HDTs trajectories, surpassing the capabilities of ground-based GPS. This system enables the creation of high-resolution emission inventories for HDTs.^4^ In non-road sectors, including shipping, the widespread use of the automatic identification system (AIS) has shifted emission inventory calculation methods from static to dynamic, offering a more detailed spatiotemporal analysis of ship CO_2_ emissions, from local ports to a global scale.^21^^,^^22^ AIS data may occasionally experience signal loss or anomalies due to satellite disruptions, equipment maintenance, or transmission issues. In aviation, growing concern over high-altitude emissions and advances in communication, navigation, and surveillance have led to the use of automatic dependent surveillance broadcast (ADS-B). This open data source allows precise emission characterization during different flight stages using time, longitude, latitude, and altitude.^23^^,^^24^^,^^25^ AIS and ADS-B have higher deployment rates than vehicle position systems, with AIS being installed on nearly all types of ships. This broad coverage allows researchers to analyze global dynamics in shipping and aviation, such as assessing the spatial variation of coronavirus disease 2019 (COVID-19) impacts (Figures 3A and 3B).^26^^,^^27^

There are also on-road monitoring technologies that provide both real-time pollutant concentrations and GPS data, enabling emissions to be calculated directly from recorded concentrations rather than relying on empirical models. One such system, the portable emissions measurement system (PEMS), collects pollutant concentrations through probes connected to the vehicle tailpipe, along with GPS data, and is widely used to determine operational conditions and emission levels on a second-by-second basis.^29^^,^^30^^,^^31^ Similarly, plume-chasing technologies measure real-time emissions by trailing a target vehicle or ship with a lab-equipped vehicle or trailer.^32^^,^^33^ While effective for monitoring emissions,^34^^,^^35^^,^^36^ their widespread application across entire fleets and the creation of comprehensive emission inventories are limited by time and cost constraints. Therefore, trajectory signals from these technologies remain underutilized. Originally intended to detect emissions exceeding standards, the on-board diagnostic (OBD) system has advanced to record operating conditions, engine parameters, and exhaust concentrations, aiding in emission inventory development.^37^^,^^38^^,^^39^ With the transition from Euro 6 to Euro 7 standards, on-board monitoring systems are expected to complement OBD systems, ensuring real-world emission compliance.^40^^,^^41^

### Traffic profile measurements

Traffic flow, pollutant concentrations, and related data are commonly measured through non-on-board technologies. These methods, targeting vehicles, roads, ships, sea surfaces, aircraft, and atmospheric layers, facilitate indirect analysis of real-world emissions and their spatiotemporal distribution.

Remote sensing, developed by the University of Denver,^42^ is the most widely used vehicle-oriented technology, with datasets available on the Fuel Efficiency Automobile Test website (https://digitalcommons.du.edu/feat/). This roadside method measures pollutant-to-CO_2_ ratios in exhaust but cannot directly quantify pollutant concentrations. It is primarily employed for regulatory purposes, such as monitoring fleet emissions^43^^,^^44^ or identifying high-emitting vehicles.^45^^,^^46^ Additionally, roadside remote sensing data are utilized to develop emission factors and models,^47^^,^^48^ primarily for common pollutants rather than GHGs. Another method, black smoke capture technology, employs image processing to detect harmful substances in vehicle exhaust. By analyzing colors and optical features, it identifies emissions, with a focus on black smoke as a key pollution indicator. Given the uncertainties in sensors and image processing algorithms, black smoke capture technology is primarily employed to identify high-emitting vehicles.^49^ However, public datasets on vehicle black smoke emissions are limited due to high deployment costs. An example is the Smoke Vehicles Computer Vision Project (https://universe.roboflow.com/alpha-ai-nwrrb/smoke-vehicles/model/3?webcam=true), which offers black smoke segmentation data and online testing tools.

Road-oriented technologies, now integral to ITS, are categorized into magnetic-frequency-based methods (such as underground loop detectors), wave-frequency-based methods (such as remote traffic microwave sensors and radio-frequency identification), and video monitoring. These systems, installed on key roads, gather data on speed, traffic volume, and lane occupancy, facilitating urban-scale emission allocation, major road emission estimates,^28^^,^^50^ and the identification of emission hotspots.^51^ While ITS lacks the detailed vehicle data, such as trajectory systems, it offers the widest coverage among on-road traffic data sources, enabling analysis of activity changes in road transport (Figure 3C).^28^ Ambient measurements, though unable to directly quantify emissions from individual vehicles or fleets, capture road pollutant concentrations with low cost and high temporal resolution.^52^^,^^53^^,^^54^^,^^55^ When combined with receptor modeling, these measurements can attribute pollutant concentrations to mobile sources, supporting the estimation of their emissions.

### Satellite observations

Advances in satellite technology have greatly improved global GHG monitoring and the understanding of anthropogenic CO_2_ emissions. Early satellites, such as AQUA and ENVISAT (both launched in 2002), pioneered atmospheric chemistry studies. Later missions, including AURA (2004), with enhanced spatial resolution, and METOP-A (2006), featuring the infrared atmospheric sounding interferometer, further advanced CO_2_ monitoring capabilities. Launched in 2009, GOSAT was the first hyperspectral satellite focused on atmospheric CO_2_ detection, providing global CO_2_ and CH_4_ data for at least 5 years. In 2018, GOSAT-2 enhanced the precision and provided more cloud-free measurements. Since 2009, the US Orbiting Carbon Observatory series (OCO-2, 2014, and OCO-3, 2019) has delivered high spatial resolution, despite longer revisit intervals. Researchers have used OCO-2 data to create multi-annual gridded CO_2_ datasets.^56^^,^^57^ In 2016, China launched TANSAT, achieving advanced levels in high-resolution atmospheric trace gas detection. Future missions, including France’s Microcarb and the France-Germany MerLin collaboration, will further enhance global GHG monitoring capabilities.

Emission detection using CO_2_ satellites varies by study scale. At regional or national levels, inverse models, such as variational data assimilation (4D-Var) and ensemble filtering, are typically used to optimize bottom-up emission inventories by minimizing the discrepancy between observed and modeled concentrations from chemical transport models (CTMs).^58^^,^^59^ These approaches often incorporate other satellite observations, such as NO_2_ column densities from OMI and TROPOMI with improved spatiotemporal resolution, to inverse CO_2_ emissions by first estimating NO_x_ emissions and converting them into CO_2_ emissions using CO_2_ and NO_x_ emission factors.^60^ This method has resulted in a CO_2_ emission product derived from TROPOMI NO_2_ observations.^61^ At the city scale, Lagrangian-based inversion methods are commonly employed for flux estimation,^62^^,^^63^ as they require fewer simulations and provide greater sensitivity to upwind sources compared with Eulerian approaches. For point sources, emissions can be directly estimated from satellite observations and wind data using methods such as Gaussian plume fitting or mass-balance approaches. These techniques, independent of CTM simulations,^64^^,^^65^ offer reduced computational costs and are more effective in estimating short-term carbon emission variations compared with bottom-up models that depend on annual updates.

The estimation of transportation CO_2_ emissions predominantly relies on 4D-Var approaches due to the insufficient temporal-spatial resolution of current CO_2_ satellite data, which cannot accurately detect dynamic emission plumes in complex, polluted environments, particularly over land where large power or industrial sources dominate. Consequently, bottom-up emission inventories are crucial for providing corrective prior information. In the aviation sector, emissions are relatively small, and satellite observations, primarily focused on the troposphere, struggle to capture the impact of aircraft emissions, which occur mainly in the stratosphere. By contrast, satellites are more effective for estimating shipping CO_2_ emissions, as vessels operate in open seas with minimal other anthropogenic sources. Examples include CTM-based global emission inventories^59^ and non-CTM methods for port emissions.^66^ To broaden satellite applications for estimating CO_2_ emissions across more transportation sectors, improving satellite data quality, especially temporal-spatial resolution, is crucial.

## AI

AI is a field focused on enabling computers to perform tasks that require human intelligence. A subset of AI, ML enhances computer systems by learning patterns from data, while DL, a specialized form of ML, uses deep neural networks to represent complex data patterns. These methods have become powerful tools for data mining. Advances in detection and characterization technologies have significantly expanded transportation data volume, driving the adoption of AI. Common ML models are now widely supported by libraries in Python, R, and MATLAB, with PyTorch and TensorFlow being the most popular DL frameworks.

**Figure 4:**
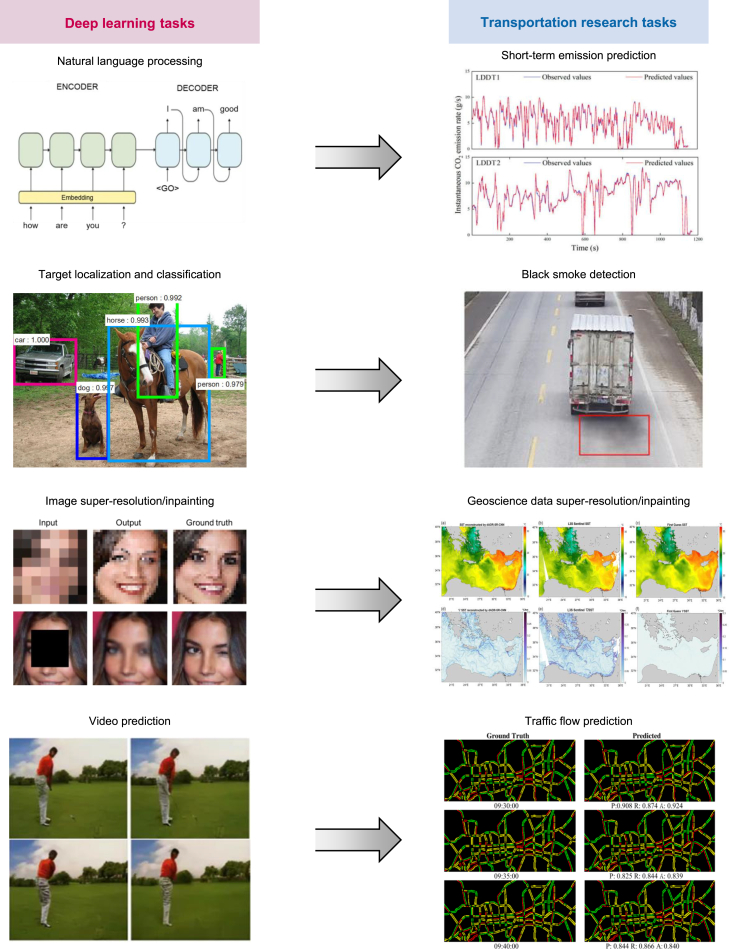
Applications of classical deep learning for transportation problems Images for short-term emission prediction, geoscience data super-resolution/inpainting, and traffic flow prediction are from Li et al.,^67^ Fanelli et al.,^68^ and Ranjan et al.^69^

AI models are generally classified into discrete models for classification and continuous models for regression. While ML is widely used in transportation, DL remains in its nascent stages but offers great promise for addressing challenges such as classification, anomaly detection, regression, and state predictions involving spatial or temporal dependencies. The similarity between the data types typically processed by classical DL applications and those in transportation and Earth sciences underscores the potential for incorporating DL into traffic science. As depicted in Figure 4, classification and regression are key challenges common across various fields, such as natural language processing, computer vision, and transportation science. This section outlines the primary AI applications in transportation emissions, categorized by transportation big data types.

### Applications in transportation emissions

The collection of operational data from on-board measurements, including PEMS, plume chasing, and OBD, has revealed discrepancies between empirical data and theoretical model predictions. Research indicates that engine-related factors,^70^^,^^71^ along with external variables, such as road gradient,^72^ road type,^73^ and atmospheric temperature,^74^ significantly influence vehicle emissions and are often overlooked in traditional models.^70^^,^^71^ Studies relying on mathematical statistics and linear regression for single-factor correlation analysis often overlook the complex interactions influencing mobile source emissions. This limitation has spurred the adoption of ML methods paired with interpretable models, such as ensemble tree algorithms, like random forest^75^ and gradient boosting machine,^76^ valued for their clarity through treelike flowchart representations. These models quantify each factor’s contribution to real-world vehicle emissions, offering critical insights to enhance empirical models. Artificial neural networks (ANNs) are also widely used, providing superior predictive accuracy compared with tree-based models but lacking interpretability.^77^^,^^78^^,^^79^ Recent innovations, such as the Shapley additive explanations^26^ framework, have improved ML interpretability by assigning importance values to features and being broadly applicable. In addition, some studies estimate fuel consumption to derive emission predictions.^80^^,^^81^^,^^82^

AI models are also applied to time-series prediction using on-board measurements. Mobile source emissions display long-term temporal dependencies, and delays between emission measurements and engine conditions can cause persistent errors despite time alignment. As a result, relying solely on current data may be insufficient for accurate predictions. Recurrent neural networks (RNNs) address this issue by leveraging previous states to inform current state learning, making them well suited for time-series data.^83^ RNNs are limited by the vanishing and exploding gradient problem, which hinders their performance on long time-series data. To overcome this, long short-term memory networks (LSTMs)^84^ and other RNN variants were developed. LSTMs are highly effective in capturing sequential dependencies, making them particularly useful in applications such as natural language processing. In atmospheric science, LSTMs have been used for real-time air pollution^85^ and carbon emission predictions.^86^ Similarly, for vehicle emissions, LSTMs, their variants, and hybrid models have been employed for emission forecasting.^67^^,^^87^^,^^88^

Trajectory and road network monitoring data are used to develop emission inventories with spatiotemporal attributes or supplement existing ones. However, widespread sensor deployment across road networks is prohibitively expensive, resulting in geographic sparsity, and trajectory data also often face signal loss and quality issues. AI approaches addressing these challenges include traffic flow prediction,^89^ data reconstruction,^90^ trajectory prediction,^91^ and transportation mode recognition.^92^^,^^93^ These studies leverage ML techniques to reframe ITS data and incorporate them with emission models, producing high-resolution emission inventories. In addition, large-scale ITS datasets have been directly used to predict traffic-related CO_2_ emissions.^94^^,^^95^

With the evolution of data characterization from one-dimensional to more complex, high-dimensional forms, big data offer a more detailed understanding of transportation modes, allowing for in-depth analysis of intricate spatial relationships. In two-dimensional data, processes at specific points are often impacted by factors related to the system’s state that are not directly observable. For instance, determining if pollutant levels, such as vehicle black smoke, exceed permissible limits cannot be based solely on the local pixel values of the smoke. It also necessitates accounting for surrounding pixels, such as those representing the background or shadows. Traditional ML methods rely on handcrafted domain-specific features,^96^ such as terrain shapes and image textures, to incorporate spatial backgrounds and address temporal dependencies. These features serve as inputs for ML tasks, such as localization, classification, and detection. While handcrafted features offer interpretative control, they are limited by time-consuming, domain-specific processes and suboptimal utilization of spatial dependencies.

Convolutional neural networks (CNNs) in DL models excel at automatically identifying complex patterns and relationships. For classification tasks, CNNs are widely employed to detect black smoke from vehicles in surveillance footage,^97^^,^^98^ improving the efficiency of automatic detection for high-emission vehicles. Traditional object detection models, such as Faster R-CNN^99^ and YOLO,^100^ are suitable for interdisciplinary research. In regression tasks, CNNs are primarily applied to geoscientific data, offering essential data for CO_2_ emission calculations rather than directly detecting emissions. Two primary applications are image super-resolution and inpainting. As noted, the current spatial resolution of CO_2_ satellite observations is inadequate for isolating the impact of traffic-related emissions. Achieving higher resolution is essential to address this limitation. Image super-resolution involves generating high-resolution images from low-resolution ones and has been applied to various remote sensing datasets, including optical satellite^101^ and climatological data,^68^^,^^102^ particularly for CO_2_ satellite observations with significant missing values. Image inpainting focuses on filling missing image segments and has been primarily used in photographs, paintings, and satellite images related to geoscience.^103^^,^^104^ For these regression tasks, models utilizing generative adversarial networks (GANs),^105^ such as SRGAN for super-resolution^106^ and the GAN-based model for inpainting,^106^ are recommended. However, the use of super-resolution and inpainting techniques for CO_2_ satellite observations is limited due to a lack of high-resolution, complete CO_2_ datasets for training.

In addition, DL extends beyond computer vision to natural language processing by combining CNNs with LSTMs. A notable example is predicting human behavior in videos, which shares significant similarities with transportation problems that involve strong spatiotemporal dependencies, such as detecting suspected black smoke across multiple frames^107^ and forecasting dynamic traffic flow in road networks.^69^^,^^108^^,^^109^

### Challenges and frontiers

The use of big data and AI in transportation encounters challenges related to algorithms, data, and computing costs. Current algorithms need enhancements in adaptability and interpretability to manage complex patterns. Reliable big data are crucial for model construction, validation, and optimization. In addition, efficiently processing vast data within limited computational resources remains a significant challenge.

#### Algorithm

AI models may closely match observed data but can produce physically inconsistent or unreliable predictions due to extrapolation or observational bias. Traditionally, physical modeling and AI have been seen as separate fields with distinct methodologies. Physical models provide direct interpretability and the ability to extrapolate beyond observations, while data-driven approaches can uncover hidden patterns across various datasets. In practice, these methods often complement each other, and the integration of physical models with AI is gaining increasing recognition.^110^^,^^111^ For instance, Liu et al. enhanced the MOVES model by incorporating a random forest trained with PEMS data, thus reducing emission rate biases and enabling its application in other countries.^75^ By contrast, Seo et al. focused on AI models, training an ANN using engine parameters from a vehicle dynamics model, constrained by real driving emission data.^78^

Enhancing prediction accuracy is essential but insufficient. While AI models excel in complex tasks, their “black-box” nature complicates interpretation. Due to the complexity of the environment-activity-emission nexus, tracing model decisions to their assumptions is challenging. However, a detailed understanding of model decision-making can be achieved by focusing on three aspects: pre-modeling, modeling-level, and post-modeling interpretability methods. The first two categories involve data preprocessing to analyze data distribution or designing inherently interpretable models, both aiding in optimal modeling solutions. The third category addresses DL models by employing techniques such as hidden layer analysis,^112^ agent-based modeling,^113^ and sensitivity analysis.^114^

Striking a balance between model performance and interpretability is essential. For traffic flow prediction, the primary emphasis is on achieving high prediction accuracy, as these data are typically used to construct emission inventories through classical emission models. However, in emission predictions based on operational conditions, while accuracy remains important, model interpretability becomes critical for understanding the factors influencing emissions.

#### Big data

AI models, with their complex and deep architectures, demand extensive ground-truth data for development.^115^ Unlike applications in chemistry, medicine, or biology, transportation science faces challenges with data volume, quality, and balance, limiting the scope of deep integration. Addressing these limitations requires extensive research. To leverage data challenges in transportation science, it is essential to develop standardized annotated databases and establish a comprehensive knowledge-based sample library encompassing diverse attributes for each sample. Key obstacles include the scarcity of openly accessible datasets and the lack of long-term collaborative sharing frameworks. While thousands of studies explore AI applications in transportation, access to raw data remains limited. While satellite observation data are publicly available, they are not tailored to the needs of transportation science. The limited availability of shared data significantly restricts the full potential of AI in transportation science.

Enriching and standardizing annotated databases through the creation of a comprehensive, attribute-rich sample library is essential, as inconsistencies in data collection methods, preprocessing techniques, and reference systems often lead to systematic errors, significantly complicating the data preparation process. Addressing these “data silos” and ensuring seamless integration demand coordinated efforts from governments and relevant stakeholders. In addition to securing high-quality data for current AI methods, it is crucial to innovate AI techniques to adapt to dynamic datasets. Given AI’s dependence on data, algorithms and applications must evolve with shifting data characteristics. For instance, transfer learning enables models to operate effectively when training and test data differ, eliminating the need for complete retraining in the target domain. Just as the growth of DL in computer vision was fueled by high-dimensional data, continuous advances in AI methodologies remain vital.

#### Computational costs

The increasing computational demands of processing transportation data, characterized by their vast spatiotemporal scale, present significant challenges. With global data volumes rapidly expanding, daily analysis will soon involve managing petabyte-scale datasets. For instance, traffic flow data in megacities, such as Chengdu, China, now exceed hundreds of millions of entries per day. Similarly, high-resolution satellite imagery used in transportation analysis imposes an even greater computational burden on AI systems, further complicating data processing.

High-performance and distributed computing offers a direct solution by leveraging local or geographically dispersed resources for computational tasks. Consequently, these approaches may surpass the capabilities of transportation science researchers. An alternative is lightweight DL, which improves computational efficiency by simplifying model complexity and reducing parameters. Techniques such as pruning, quantization, knowledge distillation, and network optimization reduce computational and storage demands while preserving model performance, making them ideal for resource-limited devices.^116^^,^^117^

## Discussion

The integration of transportation big data and AI will transform transportation systems and advance our understanding of transportation CO_2_ emissions. Advances in big data technologies offer a robust foundation for emission modeling and prediction. Physical models offer theoretical frameworks and parameters, while AI models, through pattern recognition, refine these models, improving predictions to better reflect real-world conditions.

To improve the spatiotemporal resolution of emission inventories, AI models in transportation should leverage expansive foundational big data, particularly ITS data. AI technologies can process or reconstruct these datasets to uncover richer traffic dynamics and effectively support the development of emission inventories with higher spatiotemporal resolution. Simultaneously, satellite technology offers the potential for large-scale CO_2_ emission estimation beyond traditional ITS data coverage. However, high-quality satellite data reconstruction requires advanced AI capabilities. By embracing the latest advancements in computer science and fostering interdisciplinary innovation, the full potential of big data can be realized for high-resolution insights into transportation emissions.

The use of big data and AI in transportation science offers both challenges and opportunities for innovation. Interdisciplinary collaboration is key to advancing technology and facilitating data sharing. Addressing privacy and security concerns, along with establishing technical standards, is essential for building trust in AI and big data applications. Unified standards will ensure system interoperability, driving efficiency and intelligent integration within the industry.

## Acknowledgments

This research was supported by the 10.13039/501100001809National Natural Science Foundation of China (grant nos. 42325505 and U2233203 to H.L.), the 10.13039/501100012166National Key Research and Development Program of China (grant no. 2022YFC3704200), the Tsinghua University Initiative Scientific Research Program, the Tsinghua University-Toyota Research Center, and the International Council on Clean Transportation.

## Author contributions

Z. Luo and H.L. conceptualized the article. All authors wrote the original draft and reviewed and edited the article. H.L. provided funding acquisition.

## Declaration of interests

The authors declare no competing interests.
